# Long-Term Antibiofilm Efficacy of Slippery Covalently Attached Liquid-like Surfaces in Dynamic and Static Culture Conditions

**DOI:** 10.1021/acsabm.5c00294

**Published:** 2025-06-09

**Authors:** Yufeng Zhu, Glen McHale, Hernan Barrio-Zhang, Rui Han, Gary G. Wells, Hongzhong Liu, Rodrigo Ledesma-Aguilar, Waldemar Vollmer, Nicholas Jakubovics, Jinju Chen

**Affiliations:** 1 School of Medicine, 12474 Shanghai Jiao Tong University, Shanghai 200127, China; 2 Institute for Multiscale Thermofluids, School of Engineering, 67387 University of Edinburgh, Edinburgh EH9 3FB, U.K.; 3 Department of Materials, 5156 Loughborough University, Loughborough LE11 3TU, U.K.; 4 School of Mechanical Engineering, Xi’an Jiaotong University, Xi’an 710054, China; 5 Institute for Molecular Bioscience, The University of Queensland, Brisbane 4072, Australia; 6 Centre for Bacterial Cell Biology, Biosciences Institute, 5994 Newcastle University, Newcastle upon Tyne NE2 4AX, U.K.; 7 School of Dental Sciences, Faculty of Medical Sciences, 5994 Newcastle University, Newcastle upon Tyne NE2 4BW, U.K.

**Keywords:** antibiofilm, slippery covalently attached liquid-like surfaces, surface wetting, liquid-infused surface, silver nanoparticles

## Abstract

This study explores the antibiofilm potential of slippery covalently attached liquid-like surfaces, revealing their remarkable ability to inhibit biofilm formation over extended periods, regardless of their hydrophobic or hydrophilic nature. We engineered permanently bound liquid-like solid surfaces with exceptional slipperiness, defined by ultralow contact angle hysteresis, and assessed their effectiveness against two nosocomial pathogens, (PAO1) and (FH8). These surfaces achieved a 3–5 order of magnitude reduction in biofilm formation compared to polydimethylsiloxane under both static and dynamic culture conditions over 14 days. Impressively, both the hydrophobic and hydrophilic slippery liquid-like solid surfaces significantly outperformed the widely used antimicrobial coatings containing silver particles in the long term in both static and dynamic cultures. These slippery surfaces also outperformed emerging antibiofilm surfaces such as liquid-infused surfaces in extended periods of dynamic cultures. We have demonstrated that ultralow liquid–solid friction, characterized as ultralow contact angle hysteresis, is an important predictor of the long-term antibiofilm performance of both hydrophobic and hydrophilic slippery covalently attached liquid-like surfaces, particularly in dynamic cultures. This work elucidates the interfacial mechanisms and scientific principles underpinning the design of advanced antibiofilm surfaces capable of maintaining a superior performance over the long-term.

## Graphical abstract

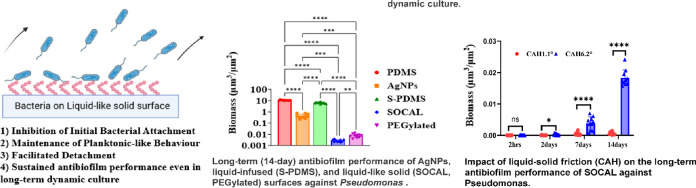

## 1 Introduction

Bacteria can grow on nearly every surface, forming complex communities, known as biofilms. Within biofilms, cells grow as multicellular aggregates encapsulated in an extracellular matrix produced by bacteria.^1^ Microbial biofilms represent a massive challenge across many sectors, from energy recovery to food security and human health. The combined economic burden across all sectors was estimated to be in excess of $5000 billion a year based on the data in 2019.^2^ For example, the annual cost of infections due to central venous catheters has been estimated at $11.5 billion globally.^3^ In the United Kingdom alone, catheter-associated urinary tract infections (CAUTIs) in hospitals incurred additional healthcare costs of £1–2.5 billion.^4^ Furthermore, a variety of other medical devices are susceptible to biofilm infections, including peritoneal dialysis catheters,^5^ tracheal prostheses,^6^ pacemakers,^7^ endotracheal tubes,^8^ dental implants,^9^ and orthopedic implants.^10^ Chemical-based approaches to combat biofilm growth using immobilization of antimicrobial agents, such as antibiotics^11^ or silver particles,^12^ are often not sustainable because they trigger antimicrobial resistance. Alternative approaches such as bioinspired textures^13^ and surface grafting with poly­(ethylene glycol) or zwitterionic polymers^14^ can be used, but their effects may not last due to adherent bacteria spreading across and masking surfaces. In these approaches, it is implicitly assumed that the underlying surface is a solid.

A recent innovation in creating surfaces slippery to liquids has been to impregnate a porous/textured solid surface with a lubricant locked into the structure by capillary forces to create a stable hemisolid/hemiliquid surface^15^ or a continuous lubricant coating.^16^ In the latter case, the slippery liquid-infused porous surface (SLIPS) mimics the slippery lubricant surface strategy of the carnivorous *Nepenthe*s pitcher plant. Such liquid lubricant surfaces, which inhibit the direct attachment of bacteria to a solid surface, have been demonstrated to be exceptional antifouling surfaces.^17^ However, performance under flow conditions where there is shear stress at the surface, such as in catheters, remains a concern due to flow-induced depletion of the lubricant. Recently, Wang and McCarthy^18^ reported similar liquid-like qualities from a simple acid-catalyzed graft polycondensation of dimethyldimethoxysilane, referred to as slippery omniphobic covalently attached liquid (SOCAL) surfaces, to covalently attach short (ca. 4 nm) non-cross-linked PDMS chains to a solid surface. This hydrophobic slippery liquid-like surface has the potential to resist biofilm formation in flow conditions in the long term, which is relevant to medical applications.^19^

It has also recently been shown that the static friction of a droplet on a surface can be regarded as a product of the contact angle hysteresis (CAH) and the normal component of the capillary adhesive force arising from the equilibrium contact angle, θ_e_, on the surface.^20,21^ Thus, the hydrophobicity/hydrophilicity of the surface determines the liquid adhesive force normal to the surface, but the chemical and physical heterogeneity of the substrate characterized by CAH translates this into friction along the surface. The resistance to biofilm formation under flow conditions of the SOCAL surface may therefore be more strongly correlated to the liquid-substrate friction than to its hydrophobicity. This motivates important questions, such as whether hydrophilic liquid-like surfaces can achieve comparable antifouling performance, whether both hydrophobic and hydrophilic liquid-like surfaces can outperform commercial antimicrobial coatings under long-term physiologically relevant conditions (e.g., 14 days in dynamic culture), and what mechanisms underlie their antifouling efficacy. Our study directly addresses these gaps, offering a comprehensive evaluation of both hydrophobic and hydrophilic liquid-like surfaces on two types of slippery covalently attached liquid-like surfaces^22–24^ (SCALS) – hydrophobic SOCAL and hydrophilic PEGylated^25,26^ – for long-term antifouling performance. By systematically comparing their efficacy under dynamic culture conditions and against clinically relevant pathogens, we provide novel insights into their potential to surpass commercial antimicrobial silver nanoparticle coatings and emerging liquid-infused surfaces. Specifically, by examining the roles of friction at the water–surface interface (quantified by contact angle hysteresis) and hydrophobicity (quantified by static contact angle) in both short- and long-term antibiofilm performances, we have uncovered the novel finding that friction plays a more critical role than hydrophobicity in determining long-term antibiofilm efficacy.

## 2 Materials and Methods

### 2.1 Material Fabrications

PDMS was prepared using a SYLGARD 184 Elastomer Kit (Dow Corning Corporation, Midland, MI) base and curing agent mixed with a ratio of 10:1 (w/w) as detailed elsewhere.^19^ The liquid-infused PDMS (S-PDMS) was prepared by immersing the cured PDMS into a silicone oil bath (Grade: 10 cSt, 0.93 g/mL, Sigma-Aldrich) and maintaining it at room temperature for 24 h, allowing oil to fully penetrate the polymer network to reach equilibrium as detailed elsewhere.^19^ The surface was gently wiped with filter paper to remove excess oil. Oil thickness (*t*) atop the surface was measured and calculated using eq 1, which assumed homogeneous thickness in all directions.^27^

$$\frac{w_{1}-w_{2}}{\rho }=(x+2t)(y+2t)(z+2t)-xyz$$ 1

where *w*_1_ is the weight of the sample before wiping, *w*_2_ is the weight of the sample after wiping, and *x*, *y*, and *z* are the length, width, and height of the wiped sample. ρ is the oil density (0.93 g/cm^3^).

Slippery omniphobic covalently attached liquid (SOCAL) surfaces were created on 25 × 75 mm glass slides using the method detailed by Wang and McCarthy.^18^ The protocol was optimized as described by Armstrong et al.^28^ The acid-catalyzed graft polycondensation of dimethyldimethoxysilane creates a homogeneous layer of nanometrically thin flexible hydrophobic polydimethylsiloxane (PDMS) chains grafted to the surface. Under acidic conditions, the methoxy groups (−OCH_3_) of dimethyldimethoxysilane hydrolyze to form silanols (Si–OH), which then form Si–O–Si bonds through polycondensation. If there is a substrate containing hydroxyl (−OH) (such as cellulose, silica, etc.), silanol can also undergo a grafting reaction with the substrate.^18^ The excessive unreacted material was then rinsed away with deionized (DI) water, 2-propanol (Fisher), and toluene (Fisher). This leads to a covalently bonded hydrophobic liquid-like layer on the surface.

To prepare PEGylated surfaces, a clean glass was exposed to an air plasma for 40 min, which deposits OH radicals to allow the PEG chains to adhere onto the surface.^25^ The sample was then immersed completely inside a solution of 2-methoxy polyethyleneoxy (6–9) propyl trimethoxysilane (Gelest), hydrochloric acid (Fisher), and toluene (Fisher), volumetric part of 1:8:10000, respectively. After 18 h of reaction, the sample was carefully removed from the solution and rinsed thoroughly with deionized water, isopropanol, and toluene. This leads to a covalently bonded hydrophilic liquid-like layer on the surface.^26^

Silver nanoparticle (AgNP) coating was synthesized on the PDMS surfaces using the protocol detailed by Kim et al.^29^ The clean PDMS was plasma cleaned for 12 min under vacuum. The samples were then immersed in 150 mL of AgNO_3_ (10 mM) (Sigma-Aldrich) solution at room temperature for 1 h. Then, 3.5 mL of NaBH_4_ (50 mM) (Sigma-Aldrich) solution was injected into 150 mL of AgNO_3_ (10 mM) solution, and it was incubated for 15 min. 150 mL of 0.0167% AgNO_3_ solution was prepared to grow Ag seeds and preheated it to 80 °C in a water bath. After 15 min of incubation, the PDMS was placed into the preheated AgNO_3_ solution for 5 min. Then, 3 mL of 1% (by weight) sodium citrate (Sigma-Aldrich) was injected as a reducing agent of silver ions. After 2 h of growth in an incubator, a uniform AgNP coating formed on PDMS. Finally, the sample was rinsed with acetone, isopropanol, and deionized water to remove the residues on the surface. Then, the sample was dried at a 37 °C incubator overnight.

### 2.2 Surface Wettability Analysis

An in-house goniometer^21,27^ was employed to measure the static water contact angle and contact angle hysteresis at ambient conditions. A drop of deionized water (volume of 8 μL) was placed on each surface. A thin needle (outer diameter: 0.4 mm), connected to a microsyringe pump, was used to control liquid movement at the droplet’s edge. Meanwhile, the contact angle was measured at the opposite edge, where the droplet maintained a near-spherical cap shape. For advancing angle measurements, the needle injected Δ*V* = 4 μL of water into the droplet at flow rates of 1 and 10 μL/min. The droplet was then allowed to equilibrate for 2 min to ensure the contact line stabilized. For receding angle measurements, Δ*V* = 4 μL of water was withdrawn at the same flow rate, followed by another 2 min of stabilization period. The difference between the advancing and receding angles are contact angle hysteresis as discussed in our previous protocol.^30^

### 2.3 Bacterial Culture and Antibiofilm Test

 (FH8) isolated from a patient with chronic rhinosinusitis at Freeman Hospital (Newcastle upon Tyne) was used.^32^ (PAO1) was also selected, which is the biofilm-forming bacterial pathogen resulting in many infections.^33^ For bacterial adhesion and biofilm formation assays, bacteria were routinely cultured in tryptic soy broth (TSB) (Melford Laboratories Ltd., UK) in a shaker at 180 rpm and 37 °C for 16 h. FH8 was diluted to OD600 = 0.2 with a spectrophotometer (Biochrom Libra S11, Biochrom Ltd., Cambridge, UK). PAO1 was diluted to OD600 = 0.01 due to its rapidly colonizing surface and overloading the system if at high concentration. For static culture (Figure 1A), samples were placed in a well plate. Three milliliters of diluted bacterial broth was added into each cell, which was then incubated at 37 °C for 2 h, 2 days, 7 days, and 14 days. For the bacterial culture lasting 7 and 14 days, half of the TSB medium needs to be replaced every 3 days to guarantee enough nutrients for bacteria to grow. At least three independent repeats were performed for each surface type.

**Figure 1:**
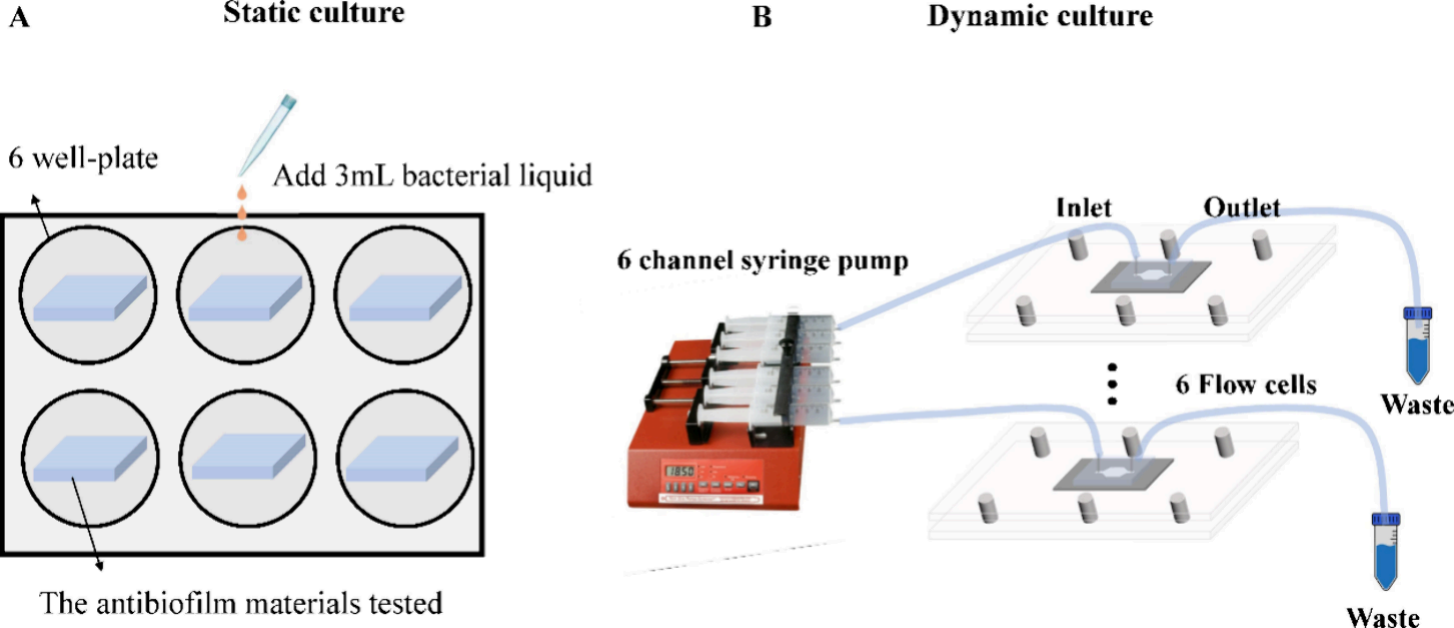
Schematic diagram of (A) static culture and (B) dynamic culture.

For dynamic culture (Figure 1B), a syringe pump together with flow cells was placed inside a 37 °C incubator. The flow cell (length = 10 mm, width = 10 mm, height = 0.1 mm) was made of PDMS and fabricated by the pattern forming on a milled acrylic block that was connected to a syringe pump. The samples were attached to the bottom of the flow cell by using a press fit. Two holes were created in the flow cell chamber: one for pumping the bacteria-inoculated broth and fresh TSB medium and another one for collecting waste. The bacterial culture was pumped into the flow chamber until the trapped air was eliminated, and then the pump ran at 37 °C for the required time. When laminar flow was well established in a parallel plate flow cell, the wall shear rate σ was given by eq 2.^31^

$$\sigma =\frac{3Q}{2{\left(\frac{h}{2}\right)}^{2}\times w}$$ 2

The wall shear stress τ_w_ is given by eq 3:^31^

$$\tau_{w}=\eta \sigma$$ 3

where *Q* was the volumetric flow rate, *h* and *w* were the height and width of the parallel plate chamber, respectively, and η was the viscosity of the culture medium at 37 °C. TSB medium had an average viscosity value of 0.7 mPa·s measured by a rheometer (Malvern Kinexus Pro+), which was used to calculate the wall shear stress.^19^

For 2 h of bacterial culture, the diluted bacterial broth was pumped into the flow cell chamber at a flow rate of 0.01 mL/min, resulting in a relative wall shear stress (τ_*w*_) of 0.007 Pa, comparable to the typical wall shear stress in a urinary catheter^34^ and ventricular catheter.^35^ For 2, 7, and 14 days of bacterial culture, diluted bacterial broth culture was supplied into the flow cell for 2 h to seed. Fresh TSB medium was then used for the required time at the same flow rate (0.01 mL/min) at 37 °C.

To evaluate the antibacterial efficacy of AgNPs coated on PDMS, bacterial viability was assessed using the FilmTracker Live/Dead Biofilm Viability Kit (Invitrogen, Life Technologies, Carlsbad, CA, USA). The bacterial killing ratio was calculated as the proportion of dead bacteria relative to the total bacterial population.

### 2.4 Statistical Analysis

Data has been represented as mean values and standard deviations. Student’s *t* test, assuming unequal variations, was applied, and *p* < 0.05 was considered statistically significant in this study.

## 3 Results

### 3.1 Surface Characteristics

Initially, a range of surfaces were produced including PDMS, silver nanoparticles (AgNPs) coated on PDMS, S-PDMS, and SCALS (SOCAL and PEGylated surfaces).

To verify the success of the AgNP coating on the PDMS surface, we used scanning electron microscopy (SEM). The AgNPs have a size of 60–140 nm (Figure S1A) and a coating thickness of about 200 nm based on the measurements of SEM with a tilted angle of 45° (Figure S1B). Energy-dispersive spectroscopy (EDS) analysis further confirmed the presence of silver and its elemental composition on individual particles (Figure S1C).

The measured contact angles (CA) and CAH for all of the surfaces are shown in Figure 2A. PDMS and AgNP-coated PDMS were demonstrated to be hydrophobic, and they had a high coupling of static liquid-substrate friction as demonstrated by high CAH values (16–21°). The S-PDMS and SOCAL surfaces exhibited hydrophobic properties, characterized by an exceptionally low coupling of static liquid-substrate friction, as indicated by their ultralow CAH values (S-PDMS: 1.61° ± 0.4°, SOCAL: 2° ± 1°). In contrast, the PEGylated surface was hydrophilic, also demonstrating ultralow static liquid-substrate friction with a CAH of 1.1° ± 0.4°. Figure 2B highlights that under continuous flow with a physiologically relevant shear stress of 0.007 Pa, oil on the S-PDMS slippery liquid-infused surface can be depleted. Previous research suggested that oil loss can impact both CAH and long-term biofilm formation.^19^

**Figure 2:**
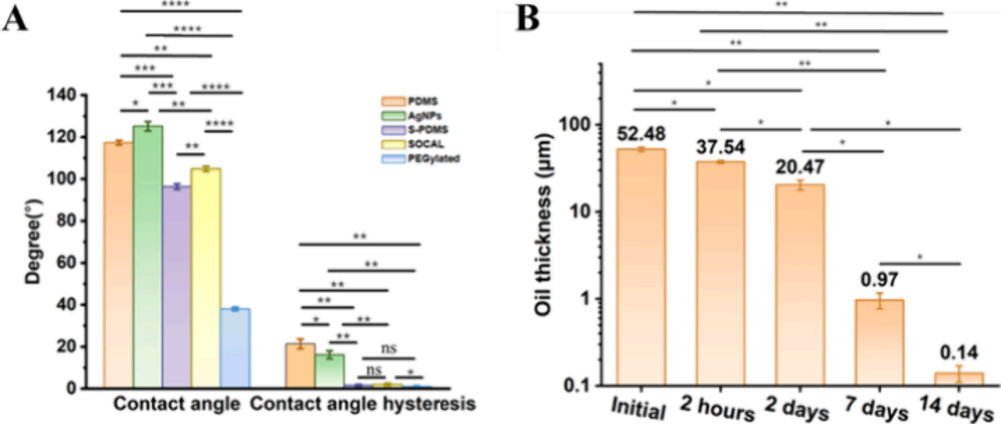
(A) CA and CAH cartograms for different surfaces and (B) oil thickness after TSB medium flow over S-PDMS for different durations. *p < 1e-3, **p < 1e-6, ***p < 1e-9, ****p < 1e-12.

### 3.2 Slippery Surfaces Outperform the Antimicrobial Agent AgNPs in Static Culture

The confocal images of and grown in static culture are shown in Figure 3A,B. Significant bacterial aggregation and adhesion were observed within 2 h, with substantial biofilm formation evident at 2, 7, and 14 days on PDMS. AgNPs could substantially reduce the adhesion of bacteria. The AgNP coating exhibited ca. 90% and ca. 80% killing ratio at 2 h against and (see Figure S2), respectively. All the other surfaces displayed reduced bacterial adhesion and biofilm formation throughout the culture periods.

**Figure 3:**
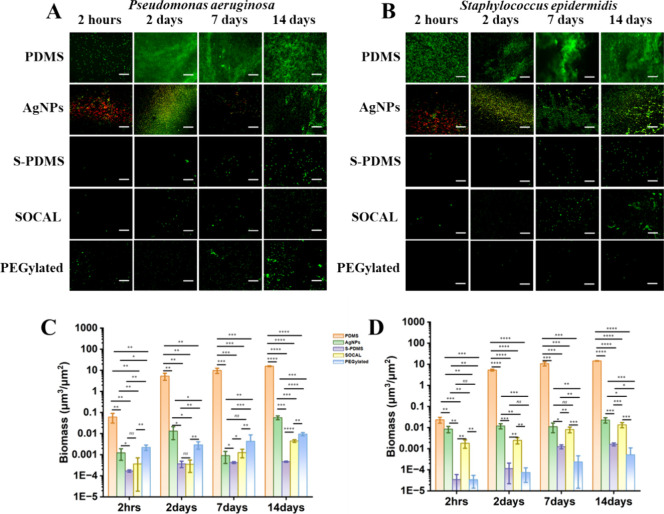
Antibiofilm activity of the surfaces against and in static culture. (A,B) Confocal microscopy images (scale bar: 50 μm). (C–D) Biomass quantification (*n* = 10). *p < 1 × 10^–3^, **p < 1 × 10^–6^, ***p < 1 × 10^–9^, ****p < 1 × 10^–12^.

Our results demonstrated a significant reduction in biofilm formation on slippery surfaces compared with PDMS controls. Over the initial 2 day to 7 day culture period, all slippery surfaces exhibited a 3–4 orders of magnitude decrease in biofilm formation compared to PDMS (Figure 3C). This effect was even more pronounced at the 14 day time point, with a 4–5 orders of magnitude reduction observed on slippery surfaces (Figure 4C). In contrast, AgNP-coated PDMS displayed a moderate biofilm reduction of 2–3 orders of magnitude throughout the 14 day culture period (Figure 3C). We also found partial detachment of the AgNP coating along with adhering biofilms after 7 or 14 days of culture, coinciding with media changes (Figure S3). This was also correlated with the decreased bacterial killing ratio (Figure S2). This phenomenon suggests weaker interfacial adhesion between the AgNP coating and the PDMS surface compared with the adhesive strength between the biofilms and the AgNPs themselves.

**Figure 4:**
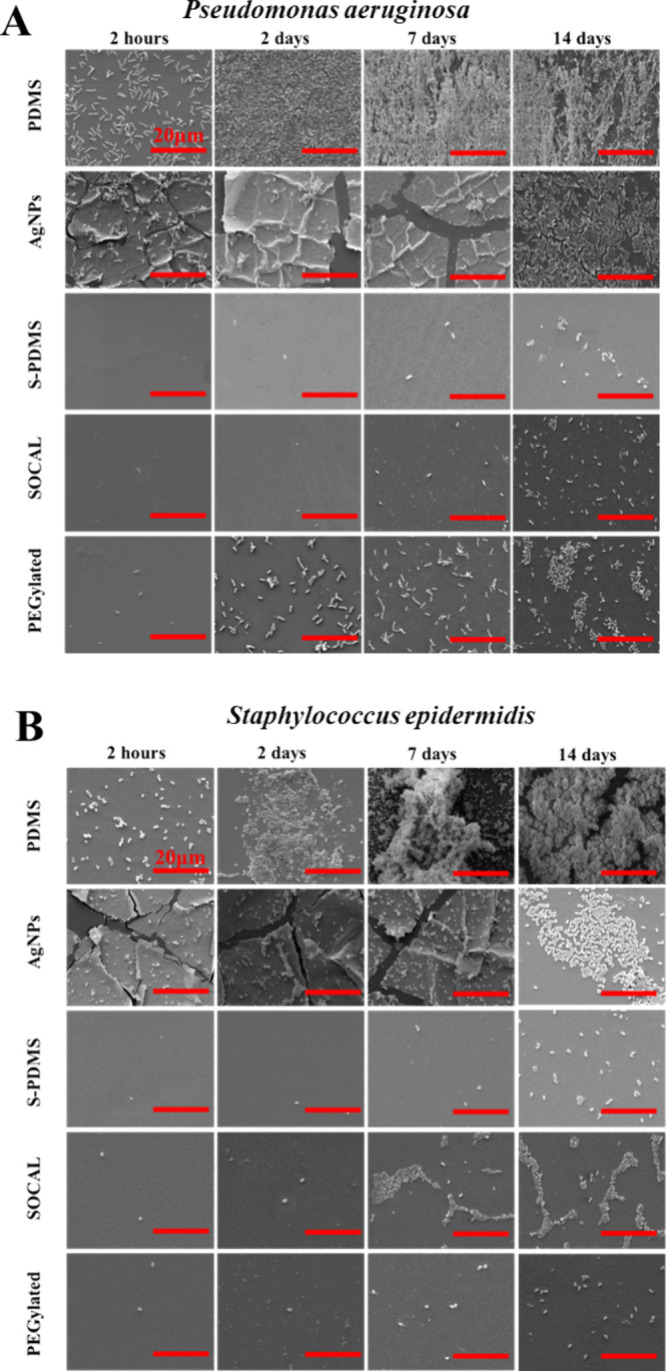
SEM images to illustrate antibiofilm performance of the surfaces against (A) and (B) after static culture. Scale bar: 20 μm.

Throughout the 14 day culture period, all slippery surfaces significantly reduced biofilm formation by 3–5 orders of magnitude compared to PDMS controls (see Figure 4D). In contrast, AgNP-coated PDMS exhibited a moderate reduction of 2–3 orders of magnitude in biofilm formation (Figure 3D).

In our comparison of two SCALS, we found that the biomass of on SOCAL was slightly lower than PEGylated at various time intervals, with differences of 6-fold, 10-fold, 3-fold, and 2-fold, respectively (*p* = 1.98 × 10^–7^, 2 h; *p* = 1.36 × 10^–6^, 2 days; *p* = 1.40 × 10^–6^, 7 days; *p* = 5.32 × 10^–8^, 14 days). S-PDMS showed similar antibiofilm performance to SOCAL at 2 h and 2 days (*p* = 0.092, *p* = 0.836) but decreased at 7 days and 14 days by 3-fold and 10-fold lower, respectively (*p* = 7.09 × 10^–4^, *p* = 1.54 × 10^–13^). In contrast, in static culture (Figure 3D), the biomass on SOCAL was higher than PEGylated across various time intervals by factors of 54-fold, 36-fold, 20-fold, and 26-fold, respectively (*p* = 8.56 × 10^–7^, 2 h; *p* = 3.13 × 10^–8^, 2 days; *p* = 8.13 × 10^–9^, 7 days; *p* = 2.24 × 10^–9^, 14 days). These observed differences between the bacterial strains may be due to their cell surface properties and the surface wettability. Normally, hydrophilic bacteria ( ^36,37^ ) have higher affinity to hydrophilic solid surfaces, while hydrophobic bacteria (^38^) have higher affinity to hydrophobic solid surfaces. These are consistent with our observations for the PEGylated and SOCAL surfaces, although we emphasize that the scale of these differences was significantly lower than the improvement in antibiofilm performance. This might not hold for liquid-infused surfaces as the bacteria interface with liquid rather than solid surfaces. While the biofilm formation on S-PDMS was very similar to that on SOCAL at 2 h and 2 days (*p* = 0.967, *p* = 0.240), it was lower at 7 and 14 days by factors of 6-fold and 8-fold, respectively (*p* = 3.33 × 10^–8^, *p* = 2.62 × 10^–5^).

High-resolution SEM images (Figure 4A,B) provide compelling evidence for the superior biofilm inhibition properties of slippery and AgNP-coated surfaces compared to PDMS. Slippery surfaces consistently displayed only sparse bacterial attachment, devoid of visible extracellular polymeric substances (EPS) throughout the 14 day culture period. The presence of EPS in the dehydrated state, as observed by SEM, was indicated by fibrous structures; a higher-magnification image is provided in the Supporting Information (Figure S4). Similar observations were also found in other papers.^14,39,40^ In contrast, PDMS surfaces exhibited significant EPS production as early as 2 days. Interestingly, AgNP-coated surfaces displayed minimal EPS production until the 14 day time point.

### 3.3 Slippery Solid Surfaces Outperform Liquid-Infused Surfaces and AgNPs in the Dynamic Culture

The confocal images of and grown in the dynamic culture with a wall shear stress of 0.007 Pa are displayed in Figure 5A,B. Significant bacterial aggregation and adhesion were evident within 2 h, with substantial biofilm formation evident at 2, 7, and 14 days on PDMS. The AgNP coating exhibited ca. 87% and ca. 65% initial killing ratios at 2 h against and (see Figure S2), respectively. All the other surfaces displayed reduced bacterial adhesion and biofilm formation throughout the culture periods.

**Figure 5:**
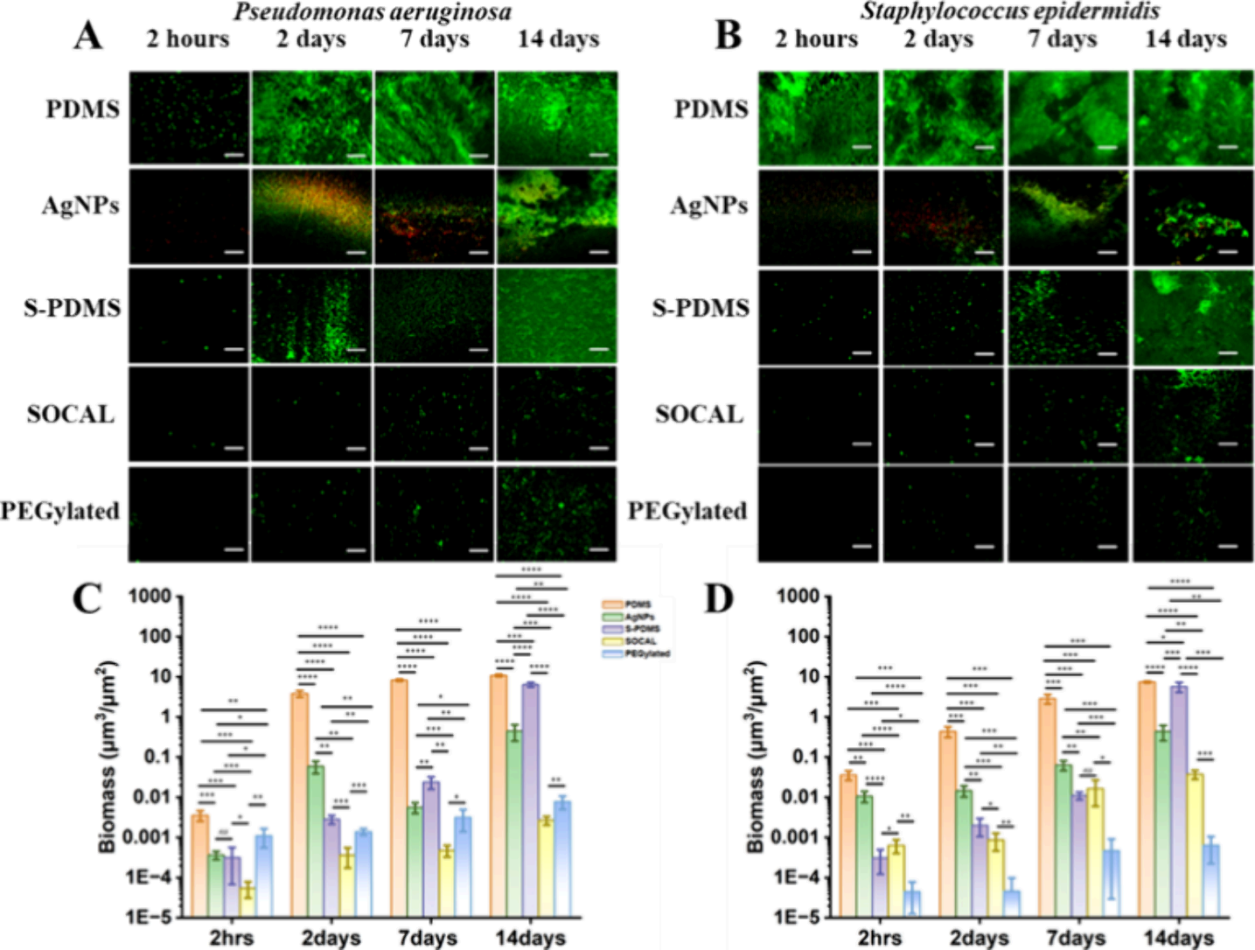
Antibiofilm activity of the surfaces against and in dynamic culture. (A,B) Confocal microscopy images (scale bar: 50 μm). (C–D) Biomass quantification (*n* = 10). *p < 1 × 10^–3^, **p < 1 × 10^–6^, ***p < 1 × 10^–9^, ****p < 1 × 10^–12^.

Over the initial 2 day culture period, all slippery surfaces exhibited a 3–4 orders of magnitude decrease in biofilm formation compared to PDMS (Figure 5C). In contrast, AgNP-coated PDMS displayed a moderate biofilm reduction of 2 orders of magnitude. After 7–14 days of flow, both SCALS surfaces still reduced biofilm formation by about 4 orders of magnitude, whereas after 7 days of continuous flow, S-PDMS only reduced biofilm formation by 2 orders of magnitude. After 14 days, the liquid-infused PDMS was only capable of reducing biofilm formation by about 40%. Such an antibiofilm property deterioration was due to flow-induced oil depletion as evidenced by oil thickness measurements in Figure 1B. Again, AgNP coating detachment was observed after long-term fluid flow, which led to significant biofilm formation after 14 days.

Throughout the 14 day culture period, all the SCALS significantly reduced biofilm formation by 3–4 orders of magnitude compared to the PDMS controls (see Figure 5D). The liquid-infused PDMS (S-PDMS) surface has comparable performance to its solid-like counterpart (SOCAL) during 2–7 days of culture. However, after 14 days of continuous flow, the liquid-infused PDMS was only capable of reducing biofilm formation by about 30% due to flow-induced oil depletion. Throughout the culture period, AgNP-coated PDMS exhibited a moderate reduction of 1–2 orders of magnitude in biofilm formation (Figure 5D).

When comparing those two SCALS in dynamic culture, we also found the biomass of on SOCAL was lower than on the PEGylated surface, while the biomass of on SOCAL was larger than on the PEGylated surface across various time intervals.

As shown in high-resolution SEM images (Figure 6A,B), the SCALS only resulted in sparsely attached bacteria with no visible evidence of EPS. In contrast, significant EPS was found on both PDMS and AgNP-coated surfaces and a 2 week-long dynamic culture S-PDMS.

**Figure 6:**
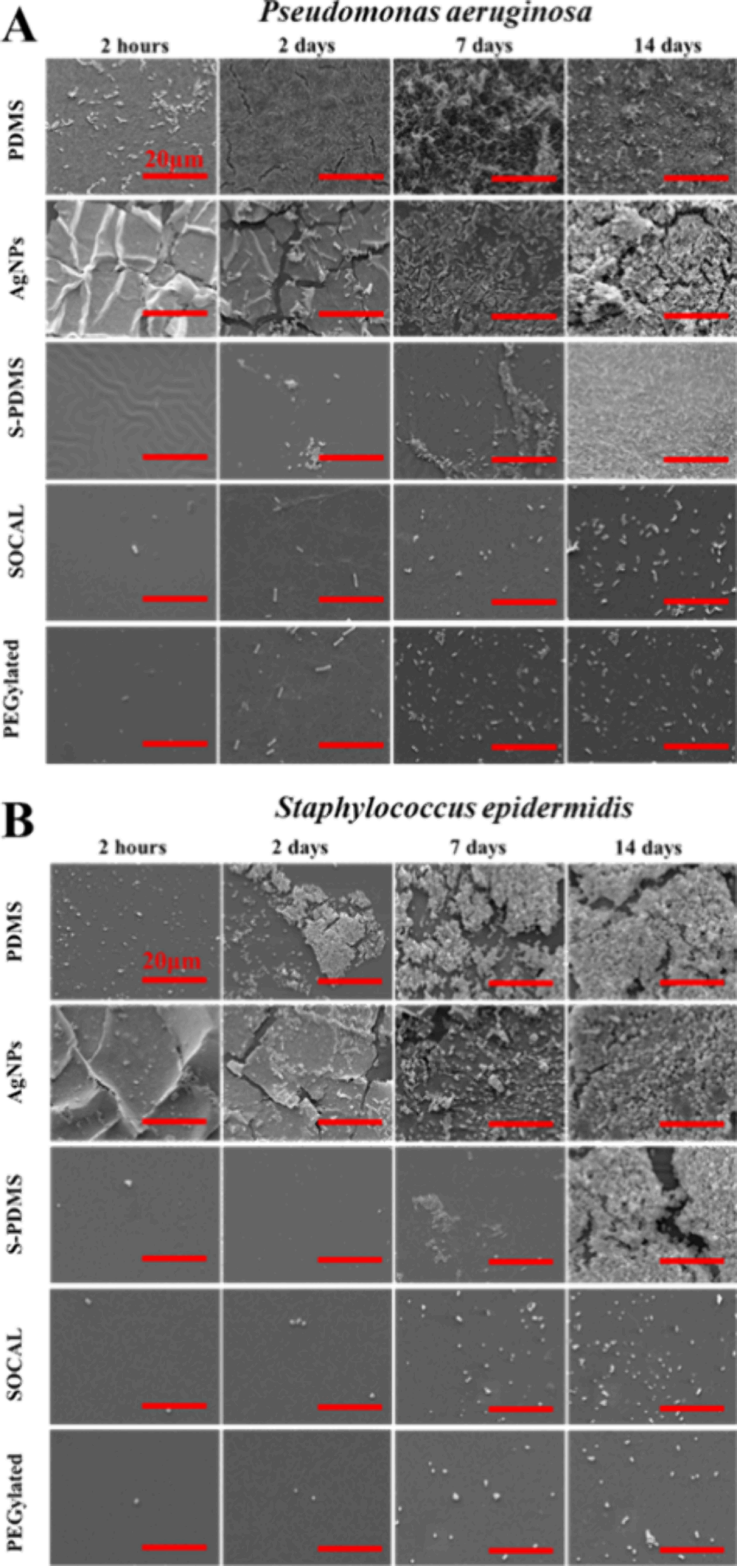
SEM images to illustrate antibiofilm performance of the surfaces against (A) and (B) after dynamic culture. Scale bar: 20 μm.

High-resolution SEM images corroborate the effectiveness of slippery and AgNP-coated surfaces in inhibiting biofilm formation (Figure 6A,B). Slippery surfaces displayed only sparse bacterial attachment, devoid of visible EPS. Conversely, both PDMS and AgNP-coated surfaces exhibited significant EPS production.

High-resolution SEM images (Figure 6A,B) provide compelling evidence for the superior biofilm inhibition properties of slippery and AgNP-coated surfaces compared to PDMS. Both slippery liquid-like solid surfaces consistently displayed only sparse bacterial attachment, devoid of visible EPS throughout the 14 day culture period. In contrast, PDMS surfaces exhibited significant EPS production as early as 2 days. Interestingly, both S-PDMS and AgNP-coated surfaces showed comparable EPS production to that of PDMS after 14 days.

## 4 Discussion

Both SCALS produced stable and long-lasting inhibition of biofilm formation in both static and dynamic conditions, whereas antibiofilm effects of AgNPs were more limited and the S-PDMS lost antibiofilm properties after extended incubation in dynamic conditions.

Our findings highlight the remarkable durability and effectiveness of SCALS (slippery, covalently attached liquid-like surfaces) in preventing bacterial biofilm formation under both static and dynamic conditions. Unlike conventional antimicrobial coatings such as silver nanoparticles (AgNPs), which exhibited transient efficacy and significant performance loss over time particularly in dynamic culture, SCALS surfaces maintained long-term antibiofilm activity—underscoring the critical role of interfacial design in resisting biofouling.

We attribute this decline of AgNP antibiofilm performance to two converging mechanisms: the gradual detachment of AgNPs from the substrate and the persistence of viable bacteria capable of initiating biofilm formation by colonizing cell debris and secreting extracellular polymeric substances (EPS). After 14 days, the efficacy of AgNP coatings was reduced by over 40-fold, suggesting a rapid loss of functional longevity under biologically relevant conditions.

Among the slippery surfaces tested, S-PDMS with a thick oil layer (∼50 μm) demonstrated a slightly superior performance in static culture. We hypothesize that the thickness of the lubricant layer disrupts bacterial mechanosensation of the underlying solid substrate, effectively suspending cells in the liquid medium and preventing the surface attachment necessary for biofilm initiation. However, in dynamic culture, the antibiofilm performance of S-PDMS with a thick oil layer decreases with time due to the flow induced oil depletion. In contrast, those liquid-like surfaces—regardless of hydrophobic or hydrophilic character—consistently suppressed biofilm formation over a prolonged period of dynamic culture because the liquid-like solid layer covalently attached to the underling substrate. There are subtle differences in surface hydrophilicity on the extent of biofilm inhibition, suggesting organism-specific interactions with the solid–liquid interface that merit further mechanistic exploration.

A key mechanistic insight emerging from this study is that ultralow contact angle hysteresis (CAH), rather than absolute contact angle or surface chemistry alone, is a dominant factor in long-term biofilm suppression. While traditional antifouling strategies have focused on reducing surface roughness or enhancing hydrophobicity, our data suggest that minimizing liquid–solid friction—a property captured by CAH—is far more predictive of antibiofilm performance, especially under prolonged or dynamic conditions.

Furthermore, based on SEM images, bacteria attached on AgNPs were still able to produce EPS, while little or no EPS was observed in biofilms on all the slippery surfaces. The initial low biomass staying below critical threshold for quorum sensing is important for inhibiting biofilm formation.^41^ Furthermore, similar to bacteria on soft hydrogels or soft polymer brush, the adhering bacteria hardly realize they are on a surface and do not change to the protected phenotype, enabling them to form a biofilm with EPS encasing on the liquid-like surfaces or liquid-infused surfaces.^42,43^

These findings suggest that both the CA and CAH of liquid-like solid surfaces influence biofilm formation. However, the long-term impact of exceptionally low CAH is particularly pronounced under dynamic culture conditions. To further investigate this, we engineered PEGylated surfaces with identical contact angles but varying CAH values (ranging from 1° to 6°). As shown in Figures S5–S6, our results demonstrate that ultralow liquid–solid friction (CAH = 1.1° ± 0.4°) substantially reduces biofilm formation over time in both static and dynamic cultures. Notably, in dynamic conditions, surfaces with ultralow CAH achieved an additional 8- to 20-fold reduction in biofilm formation compared to surfaces with a CAH of 6°. This suggests that liquid–solid friction plays a crucial role in facilitating the detachment of bacteria under flow conditions.

It is important to note that these liquid-like solid surfaces are ultrasmooth, with a roughness below 1 nm.^21,44^ While the ultrasmooth nature of these surfaces can reduce initial attachment, it typically does not prevent long-term biofilm formation. Therefore, we propose that a low liquid–solid friction is likely the dominant factor influencing antifouling performance.

We propose the following antibiofilm mechanisms for different surfaces, as illustrated in Figure 7:

1) Inhibition of Initial Bacterial Attachment: The ultrasmooth and soft nature of liquid-like solid surfaces, along with low contact angle hysteresis, effectively hinders the initial attachment of bacteria. This prevents cells from accumulating to a point where there is sufficient biomass to trigger quorum sensing.
2) Maintenance of Planktonic-like Behavior: When bacteria come into contact with liquid or liquid-like surfaces, they remain in a planktonic-like state, primarily proliferating with little to no production of EPS.
3) Facilitated Detachment: Bacterial cells are unable to form stable and strong interactions with liquid or liquid-like solid surfaces. As a result, they detach easily during growth or under the influence of gentle external forces such as physiological flow.

**Figure 7:**
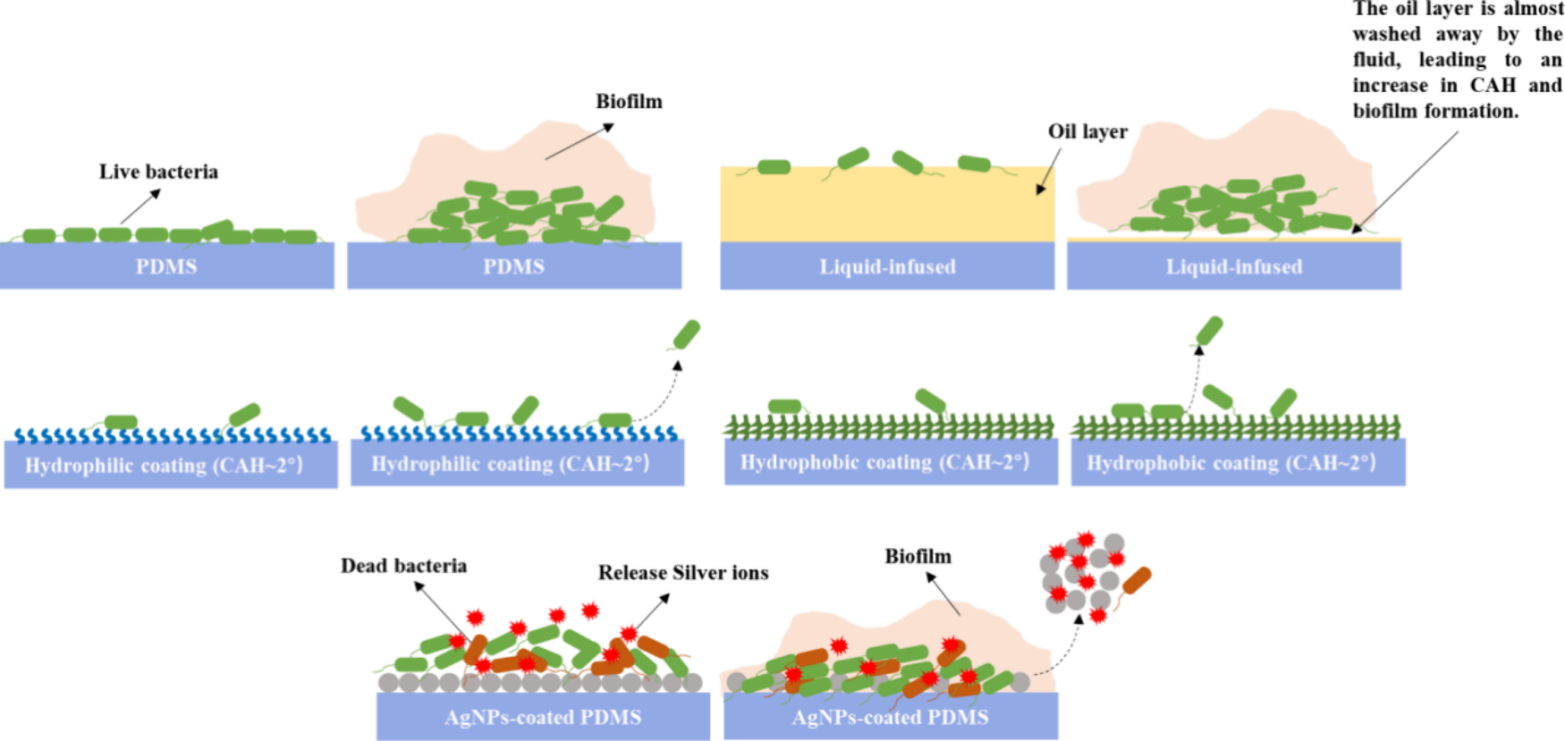
Antibiofilm mechanisms for different surfaces in dynamic culture. Green: live bacteria; brown: dead bacteria; red: silver ions; pink: biofilm with EPS; yellow: silicone oil layer.

Together, these mechanisms play a crucial role in preventing biofilm formation on slippery surfaces. This mechanism would explain why we did not observe cell clusters or biofilms on surfaces with either hydrophobic or hydrophilic liquid-like solid surfaces even after 2, 7, and 14 days of culture in static and dynamic conditions.

This work demonstrates the potential of both hydrophilic and hydrophobic slippery liquid-like solid surfaces (with CAH < 3°) for long-term prevention of biofilm formation. These surfaces significantly outperform antimicrobial surfaces that rely on bacterial killing and also surpass liquid-infused surfaces under continuous flow conditions. Notably, these findings hold promise for various medical applications.

## 5 Conclusions

This study highlights the remarkable antibiofilm potential of liquid-like solid surfaces, demonstrating their ability to effectively inhibit biofilm formation over extended periods, irrespective of their hydrophobic or hydrophilic nature. By engineering permanently bound liquid-like solid surfaces with ultralow contact angle hysteresis, we achieved a 3–5 orders of magnitude reduction in biofilm formation against (PAO1) and (FH8) compared to PDMS under both static and dynamic culture conditions over 14 days. Notably, these surfaces outperformed the widely used antimicrobial coatings containing silver particles, which rely on bactericidal effects but face limitations such as surface fouling by dead bacteria in the short term and biofilm-induced detachment in the long term. Similarly, while emerging antibiofilm solutions such as liquid-infused surfaces show promise, their performance under dynamic culture conditions diminishes due to flow-induced oil depletion. In contrast, liquid-like solid surfaces are covalently bonded to the substrate, ensuring their long-term stability and exceptional antibiofilm properties in both static and dynamic cultures.

While hydrophobicity can influence antibiofilm performance against different bacteria, it is not the predominant factor. The key lies in the nature of the liquid-like behavior of the surface (highly mobile polymer chains covalently attached to the underlying substrate), which appears ultrasmooth and chemically homogeneous. For these liquid-like solid surfaces, decreased contact angle hysteresis leads to improved antibiofilm efficacy over the long-term, especially under dynamic conditions. This work underscores the potential of liquid-like solid surfaces with ultralow liquid–solid friction (characterized by ultralow contact angle hysteresis) as a robust and promising strategy for long-term antibiofilm applications in both static and dynamic environments.

## Supplementary Material



## Data Availability

The authors declare that the main data supporting the findings of this study are available within the article and its Supporting Information. Further information and requests for resources and reagents should be directed to and will be fulfilled by the corresponding author. All data generated in this study and its Supporting Information are provided as a Supplementary Source Data file in the Supporting Information.

## Notes

Characterization of AgNP coating on PDMS; Comparative analysis of bacterial killing ratio in *Pseudomonas aeruginosa* and *Staphylococcus epidermidis* exposed to AgNPs under static and dynamic conditions; SEM imaging reveals the evolution of AgNP coating throughout the 7 day dynamic culture of *Pseudomonas aeruginosa*; SEM images revealing fibrous structure of EPS; Comparisons of initial bacterial attachment and biofilm formation for the PEGylated surface with ultralow liquid–solid friction (CAH = 1.1° ± 0.4°) and low liquid–solid friction (CAH = 6.2° ± 0.54°) of *Pseudomonas aeruginosa* in both static and dynamic culture; Comparisons of initial bacterial attachment and biofilm formation for the PEGylated surface with ultralow liquid–solid friction (CAH = 1.1° ± 0.4°) and low liquid–solid friction (CAH = 6.2° ± 0.54°) of *Staphylococcus epidermidis* in both static and dynamic culture (PDF)
