# Supplementary material for: Long-Term Antibiofilm Efficacy of Slippery Covalently Attached Liquid-like Surfaces in Dynamic and Static Culture Conditions
Source: ACS Appl Bio Mater. 2025 Jun 9;8(7):5660–9. doi: 10.1021/acsabm.5c00294 (PMC12284853; doi:10.1021/acsabm.5c00294)
Supplement: Supplementary file 1 [file mt5c00294_si_001.pdf]

# Supporting Information

# Long-Term Antibiofilm Efficacy of Slippery Covalently-Attached Liquid-Like Surfaces in Dynamic and Static Culture Conditions

Yufeng Zhu[1], Glen McHale[2], Hernan Barrio-Zhang[2], Rui Han[3], Gary G. Wells[2], Hongzhong Liu[4], Rodrigo Ledesma-Aguilar[2], Waldemar Vollmer[5,6], Nicholas Jakubovics[7], Jinju Chen[3]*

**[1] School of Medicine, Shanghai Jiao Tong University, Shanghai 200127, China.**

**[2] Institute for Multiscale Thermofluids, School of Engineering, University of Edinburgh, Edinburgh, EH9 3FB, UK**

**[3]Department of Materials, Loughborough University, Loughborough, LE11 3TU, UK**

**[4]School of Mechanical Engineering, Xi'an Jiaotong University, Xi'an 710054, China,**

**[5]Institute for Molecular Bioscience, The University of Queensland, Brisbane, Australia**

**[6]Centre for Bacterial Cell Biology, Biosciences Institute, Newcastle University, Newcastle upon Tyne, NE2 4AX, UK**

**[7]School of Dental Sciences, Faculty of Medical Sciences, Newcastle University, Newcastle Upon Tyne, NE2 4BW, UK**

## Corresponding author

Correspondence to: Jinju Chen

ORCID ID: **0000-0002-9792-6285**

***E-mail: j.chen4@lboro.ac.uk**

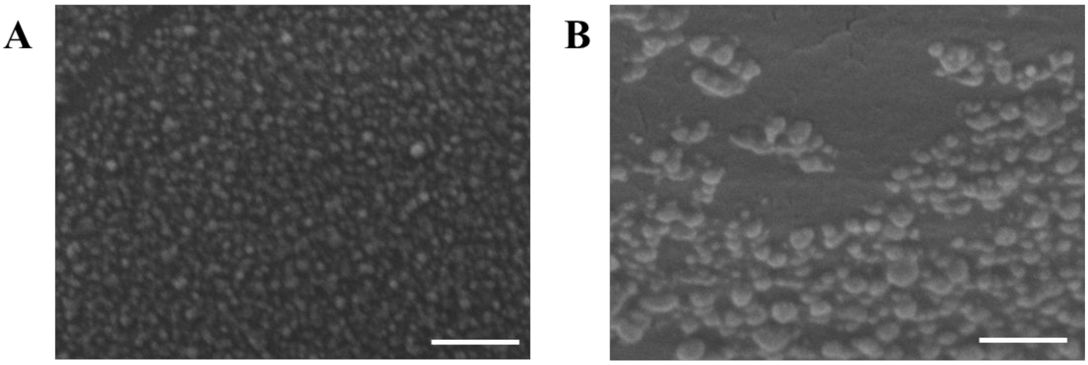


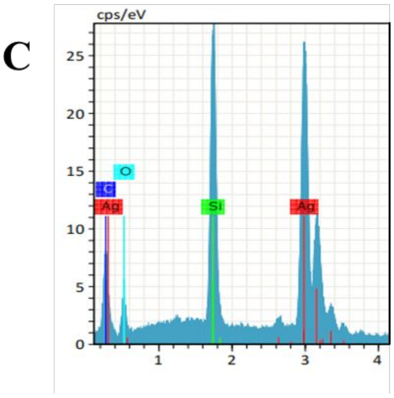


| Element | At. No. | Line s. | Netto | Mass [%] | Mass Norm. [%] | Atom [%] | abs. error [%] (3 sigma) | rel. error [%] (3 sigma) |
|---|---|---|---|---|---|---|---|---|
| Carbon | 6 | K-Serie | 5185 | 1.56 | 3.61 | 16.11 | 0.80 | 51.46 |
| Oxygen | 8 | K-Serie | 3117 | 1.74 | 4.03 | 13.49 | 0.97 | 55.80 |
| Silicon | 14 | K-Serie | 35289 | 7.52 | 17.39 | 33.17 | 1.01 | 13.39 |
| Silver | 47 | L-Serie | 74821 | 32.42 | 74.96 | 37.22 | 3.34 | 10.29 |
| | | | Sum | 43.25 | 100.00 | 100.00 | | |

**Figure S1. (A) The SEM images for AgNPs on the PDMS surface.** The single AgNP diameter ranges from 60nm to 140nm. **(B) The SEM images for AgNPs on the PDMS surface after tilted 45 degree.** The average thickness is around 200nm.**(C) EDS for AgNPs coating.** Scale bar=1um.

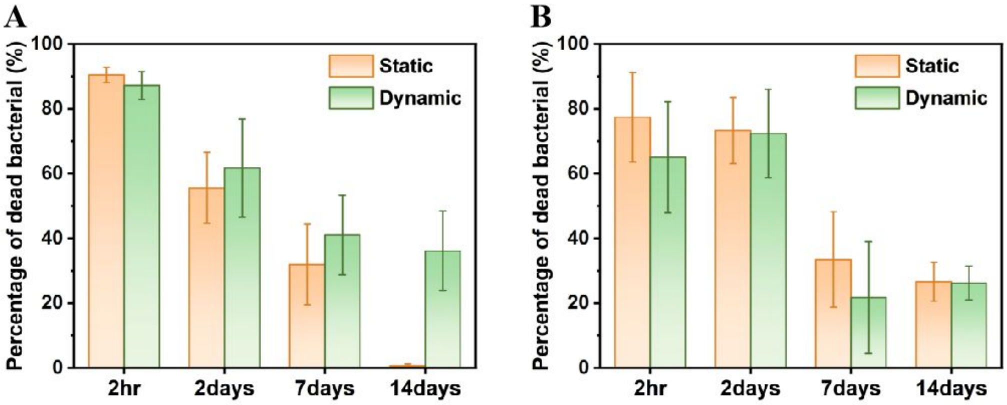


**Figure S2. The percentage of dead bacterial in both static and dynamic conditions for AgNPs (A) *Pseudomonas aeruginosa* and (B) *Staphylococcus epidermidis.*** In all cases, 10 images were analyzed for each surface from 3 independent experiments.

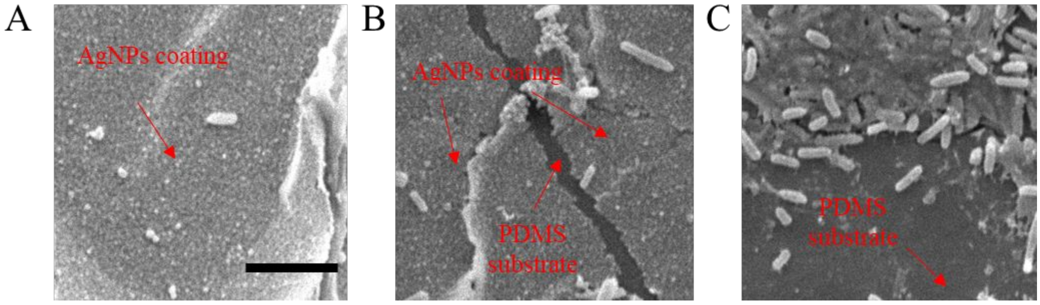


**Figure S3. SEM images showing variations in the AgNPs coating during PAO1 dynamic culture.** (A) After 2 hours of culture, the AgNPs coating remains evenly distributed; (B) After 2 days of culture, cracks began to appear on the AgNPs coating. (C) After 7 days of culture, the coating has largely detached, and bacterial adhesion has commenced. Scale bar=10μm.

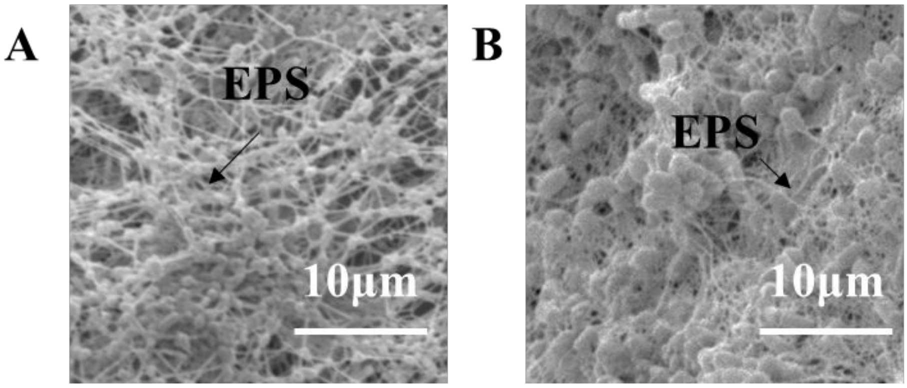


**Figure S4. SEM images showing the EPS in (A) *Pseudomonas aeruginosa* and (B) *Staphylococcus epidermidis*.**

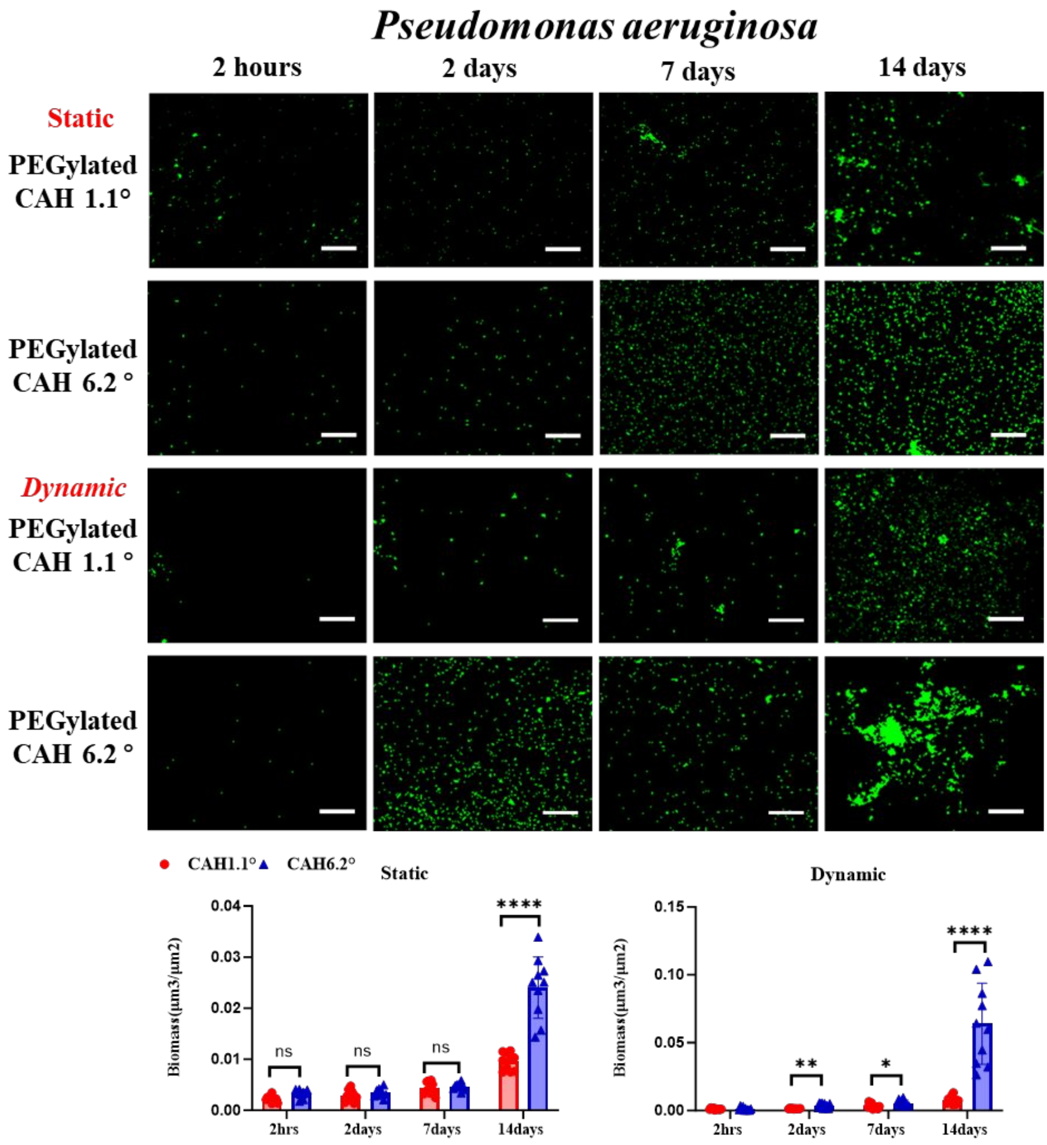


**Figure S5.** Comparisons of initial bacterial attachment and biofilm formation for PEGylated surface with ultra-low liquid-solid friction (CAH=1.1°±0.4°) and low liquid-solid friction (CAH=6.2°±0.54°) of *Pseudomonas aeruginosa* in both static and dynamic culture. scale bar: 50 µm

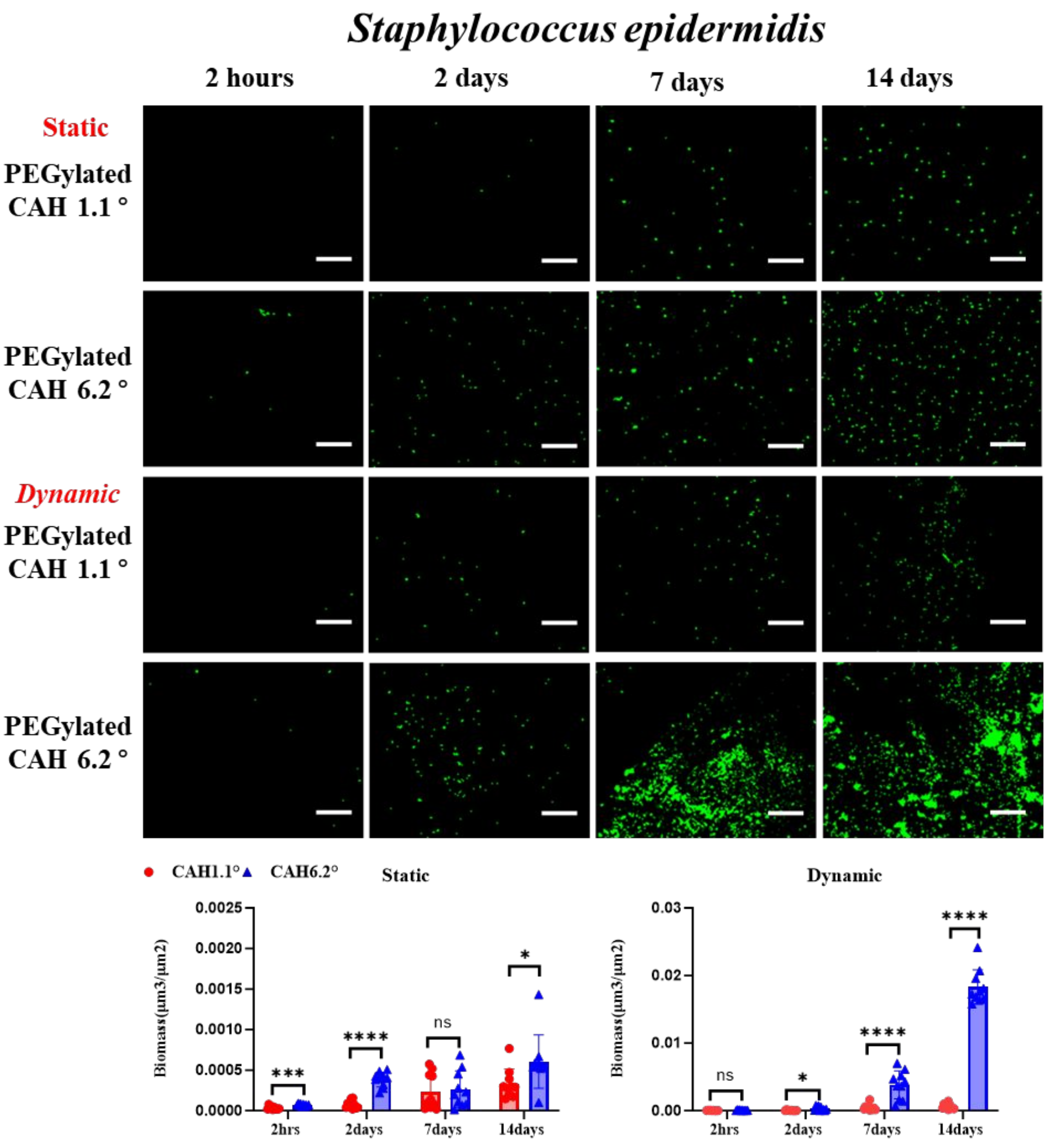


**Figure S6.** Comparisons of initial bacterial attachment and biofilm formation for PEGylated surface with ultra-low liquid-solid friction (CAH=1.1°±0.4°) and low liquid-solid friction (CAH=6.2°±0.54°) of *Staphylococcus epidermidis* in both static and dynamic culture. scale bar: 50 μm
